# Mesalamine and azathioprine modulate junctional complexes and restore epithelial barrier function in intestinal inflammation

**DOI:** 10.1038/s41598-019-39401-0

**Published:** 2019-02-26

**Authors:** Vineeta Khare, Anita Krnjic, Adrian Frick, Christina Gmainer, Mario Asboth, Kristine Jimenez, Michaela Lang, Maximilian Baumgartner, Rayko Evstatiev, Christoph Gasche

**Affiliations:** Department of Internal Medicine III, Division of Gastroenterology and Hepatology, Medical University of Vienna, Vienna, Austria

## Abstract

Disruption of mucosal structure and barrier function contribute to the pathogenesis of inflammatory bowel disease (IBD). Efficacy of therapy in IBD is based on endoscopic mucosal healing, which occurs by a dynamic interplay of epithelial cell regeneration, migration and differentiation. Both mesalamine (5-ASA) and azathioprine (AZTP) promote this process through mechanisms not clearly understood. We examined molecular pathways implicated in epithelial barrier function that were altered by 5-ASA and AZTP. Paracellular permeability induced by inflammatory mediators was mitigated by both compounds through restoration of cellular anchoring complexes. 5-ASA and AZTP induced rearrangement and membranous localization of junctional proteins and modulated genes involved in tight junctions. Intestinal organoids from wildtype-mice treated with TNF-α and IL-10- deficient-mice displayed impaired epithelial barrier with loss of membranous E-cadherin and reduced Desmoglein-2 expression. These effects were counteracted by 5-ASA and AZTP. Unlike AZTP that exhibited antiproliferative effects, 5-ASA promoted wound healing in colon epithelial cells. Both affected cellular senescence, cell cycle distribution and restricted cells in G1 or S phase without inducing apoptosis. This study provides mechanistic evidence that molecular actions of 5-ASA and AZTP on intestinal epithelia are fundamental in the resolution of barrier dysfunction.

## Introduction

Disruption of intestinal architecture associated with alterations in intestinal barrier function is a hallmark of inflammatory bowel diseases (IBD) like ulcerative colitis (UC) and Crohn’s disease (CD)^1^. The epithelial barrier is formed by intestinal epithelial cells (IEC), and barrier integrity is maintained by apical junctional complexes comprised of tight junctions (TJs) and adherens junctions (AJs). Furthermore, desmosomes connect adjacent enterocytes; and hemidesmosomes anchor the cell to the extracellular matrix. Intercellular adhesion at luminal surfaces tightly regulate epithelial permeability; while the mucosal layer produced by IEC further protects from toxic molecules, food antigens and microorganisms. A previous genome-wide association study on UC identified the CDH1 locus, which encodes E-cadherin, an integral member of AJs. This was also described as one of the susceptibility loci for colorectal cancer^2,3^. E-cadherin was also found to be mutated, with abnormal localization, in biopsies from patients with CD^4^. Genes such as CDH1,GNA12 and PTPN2 within IBD-associated loci are also implicated in mucosal barrier function^1^. Recently, the IBD susceptibility locus C1orf106 was shown to regulate stability of AJs by controlling surface E-cadherin levels through ARF6 activation^5^, substantiating the role of E-cadherin and AJs in intestinal barrier function. Pro-inflammatory cytokines like IFN-γ and TNF-α that play a central role in chronic gut inflammation, are known to cause epithelial barrier dysfunction^6^. Increased intestinal permeability by TNF-α is caused by enhancement of matrix metalloproteinases, activation of myosin light chain kinase through ERK1/2 signalling, and apoptosis^7,8^. Epithelial permeability could also be disrupted due to increased oxidative stress in IBD^9,10^. This is attributed to increased reactive oxygen species (ROS) or nitrogen radicals released from cells of the innate immune system. This is supported by the findings that mucosal tissues from patients with active disease show increased expression of nitric oxide synthase, and DNA oxidative damage marker 8-hydroxydeoxyguanosine^11,12^. Recently, our lab findings demonstrated that oxidative stress in IBD patients and in the animal model of spontaneous colitis is associated with increased DNA damage, contributing to intestinal carcinogenesis^13^. Altogether, these data suggest that chronic inflammation in IBD alters mucosal homeostasis by affecting multiple cellular pathways in intestinal epithelium.

Current IBD management uses anti-inflammatory drugs (5-ASA/mesalamine) and systemic immune modulators such as corticosteroids and thiopurines (azathioprine; AZTP and its metabolite 6-mercaptopurine, 6MP). The primary goal is mucosal healing, by suppression of inflammation and maintenance of clinical remission. These drugs are effective alone: 5-ASA in mild to moderate UC; or thiopurines in moderate to severe IBD. They may also be used in combination^14^. AZTP is considered a prodrug, which is metabolized to 6MP and other metabolites such as 6-thioguanine. Thiopurines are known to suppress immune response; and studies demonstrate that thiopurines exert their protective effects by blocking RAC1 activation in T lymphocytes and endothelial cells^15,16^. However, little is known about its effect on the intestinal epithelium. In contrast, 5-ASA is known to affect intestinal epithelial homeostasis: via inhibition of multiple pathways such as Wnt/β-catenin, ERK1/2, AKT1, mTOR, and NF-kB pathways, as well as via induction of cell cycle arrest^17^. Furthermore, it is proposed to be chemopreventive in colitis-associated cancer^18,19^. An increase in intercellular adhesion through membranous restoration of AJ proteins like E-cadherin and β-catenin is a novel proposed mechanism of 5-ASA, with direct implications on mucosal healing^20,21^. Our previous studies showed that p-21 activated kinase- 1(PAK1), an effector kinase of Rho-GTPases RAC1 and CDC42, is inhibited by 5-ASA, thereby mediating its activity^20,22^. PAK1 is a serine threonine kinase, contributing to multiple signaling pathways implicated in inflammation and carcinogenesis, and recently identified as a driver of colitis in an integrated *in vivo* multiomics approach^23,24^. Inhibition of RAC1 by AZTP or its effector molecule PAK1 by 5-ASA, underscores the role of Rho-GTPase signaling in the IBD pathogenesis. Moreover, RAC1 and CDC42 are critical players in epithelial restitution and regulation of barrier function through organization of filamentous actin in polarized epithelium^25,26^. Notably, RAC1 polymorphism is associated with UC^27^.

Although mucosal healing could be achieved by therapeutics in IBD, the underlying molecular mechanisms might differ. For example, steroids are not effective as maintenance therapies. Similarly, mucosal healing could also be achieved with AZTP, however, its molecular effects on epithelial cells are not known and thereby cannot be mechanistically compared with healing effects of 5-ASA. Hence, it is critical to identify their molecular interactions with the intestinal epithelium to elucidate the mechanistic aspects of mucosal healing. Besides, whether these drugs act through similar pathways or exhibit differential molecular effects has not been investigated in IEC. Utilizing functional assays of paracellular permeability and wound healing, we examined the underlying molecular pathways influenced by 5-ASA and AZTP towards the maintenance of epithelial barrier integrity.

## Results

### Effect of 5-ASA and AZTP on paracellular permeability

**Figure 1:**
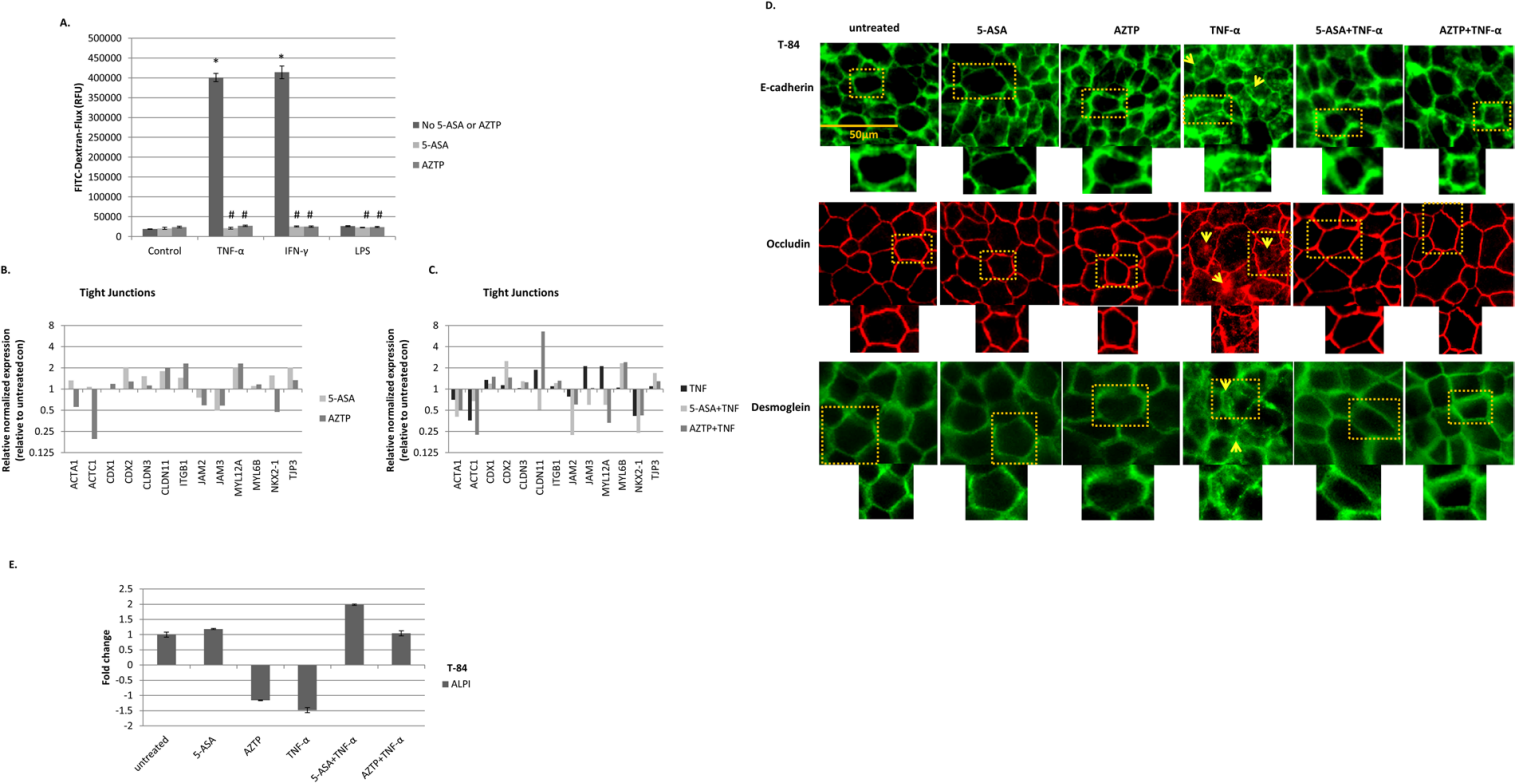
5-ASA and AZTP restore epithelial barrier. (**A**) Paracellular permeability assay using FITC-Dextran: Differentiated and polarized T-84 monolayer grown in Transwell was treated with TNF-α (10 ng/ml), IFN-γ (100U/ml) or LPS (10 ng/ml) with or without pretreatment (5 h) with 5-ASA (5 mM) or AZTP (10 μM) n = 5 each treatment. Experiments were performed in biological duplicates and repeated three times. p value (≤0.05), * con vs treatment; # vs TNF-α or IFN-γ. One-way ANOVA with multiple comparisons were done using Bonferroni’s post hoc analysis. (**B**,**C**) PCR array analysis of T-84 cells treated with 5-ASA (5 mM) and AZTP(10 μM), in the presence or absence of TNF-α (10 ng/ml) (**D**) Immunofluorescence analysis of T-84 monolayer for the representative proteins of adherens junctions; AJs (E-cadherin), tight junctions; TJs (occludin), Desmosomes (Desmoglein-2). TNF-α treatment disrupted all junctions and resulted in protein internalization as indicated by arrows. 5-ASA and AZTP restored membranous expression of these proteins. Image magnification 400x. Scale 50μm. E-cadherin and Desmoglein were visualized using Alexa Fluor 488 secondary antibody. Occludin was visualized using Alexa Fluor 568 (**E**) qRT-PCR analysis of intestinal alkaline phosphatase as a marker of cellular differentiation in T-84 cells. The experiment was performed twice.

To examine the effect of 5-ASA and AZTP on epithelial permeability, polarized T-84 monolayers were pretreated with these compounds and then treated overnight with inflammatory mediators (TNF-α, IFN-γ or LPS). A significant increase in FITC-dextran flux was observed upon TNF-α and IFN-γ treatment, which was prevented in 5-ASA or AZTP pretreated monolayers (Fig. 1A). LPS, and compounds alone did not alter permeability. Similar to AZTP, its metabolite 6MP pretreatment also reduced permeability upon TNF-α treatment (Supplementary Fig. 1A)

Furthermore, tran-sepithelial electrical resistance (TER) measurement was performed to compare the effect of the compounds in the establishment of the monolayer (Supplementary Fig. 1B). AZTP treatment increased resistance that reached highest on day 5. As was expected, TNF-α treatment delayed formation of tighter junctions (reduced TER), and both 5-ASA and AZTP counteracted this effect. TER was also measured on an established monolayer in the presence of the compounds at 24 h and 48 h time points. As observed previously, TER was reduced by TNF-α and this effect was prevented by 5-ASA and AZTP (Supplementary Fig. 1C).

Next, we performed permeability assay on a disrupted monolayer after cytokine treatments (TNF-α, IL-13). Here 5-ASA and thiopurines were used after cytokine treatments. While both 5-ASA and AZTP were effective in restoring the permeability upon TNF-α treatment, IL-13 induced permeability was not reduced (Supplementary Fig. 1D) after overnight treatment (18 h).This is likely due to differential effects of cytokines in disrupting permeability in T-84 model system.

TJs are regulated by phosphorylation of proteins by multiple kinases that affect degree of sealing^28^. We have previously reported that 5-ASA interferes with cell adhesion pathway and restores AJs through membranous localization of E-cadherin and β-catenin, without affecting total protein expression^29^. To determine the molecular targets implicated in the maintenance of TJs, a commercially available PCR array was utilized (Fig. 1B,C). Only 13 genes out of 40 showed any regulation (≥1.5 fold) in at least one of the conditions for the TJ array (Table 1). Many of the targets showed a similar trend of regulation by both compounds, albeit not to the same extent. Interestingly, increased expression of myosin light chain regulatory peptide (MYL12 A) by TNF-α was counteracted by both 5-ASA and AZTP; which could contribute to decreased permeability in the presence of these compounds. Overall 5-ASA and AZTP affected transcriptional regulation of proteins critical in regulating mucosal barrier: epithelial cytoskeletal proteins, claudins and junctional adhesion molecules (JAMs) were modulated.

**Table 1:** Differential expression of TJ genes under different experimental conditions is shown.

T-84	TIGHT JUNCTIONS PCR array	Gene Symbol	untreated	Relative normalized expression				
	Gene Name			5-ASA	AZTP	TNF	5- ASA + TNF	AZTP + TNF
1	actin, alpha 1, skeletal muscle	ACTA1	1	1.327	0.556	0.701	0.403	0.225
2	actin, alpha 2, smooth muscle, aorta	ACTA2	1	0.494	0.580	1.252	2.435	0.506
3	actin, beta	ACTB	1	1.364	1.122	0.984	1.201	1.434
4	actin, alpha, cardiac muscle 1	ACTC1	1	1.072	0.196	0.358	0.676	1.227
5	caveolin 1, caveolae protein, 22 kDa	CAV1	1	0.629	1.019	0.908	1.062	1.261
6	caudal type homeobox 1	CDX1	1	1.010	1.186	1.350	1.197	1.492
7	caudal type homeobox 2	CDX2	1	2.049	1.280	1.131	2.484	1.452
8	claudin 1	CLDN1	1	0.508	0.914	0.807	0.595	0.729
9	claudin 11	CLDN11	1	1.801	2.030	1.868	0.508	6.594
10	claudin 18	CLDN18	1	0.289	0.544	N/A	N/A	0.710
11	claudin 2	CLDN2	1	0.502	1.463	1.246	0.795	1.278
12	claudin 3	CLDN3	1	1.521	1.125	1.026	1.290	1.246
13	claudin 4	CLDN4	1	0.499	0.673	0.905	1.193	1.280
14	claudin 5	CLDN5	1	0.766	0.869	0.742	0.603	0.486
15	claudin 7	CLDN7	1	1.268	1.154	1.125	0.642	0.636
16	claudin 8	CLDN8	1	0.951	1.243	3.128	0.584	2.682
17	cold shock domain protein A	CSDA	1	0.753	1.163	1.347	0.989	1.291
18	F11 receptor	F11R	1	0.881	0.716	0.921	1.184	1.119
19	integrin, beta 1 (fibronectin receptor)	ITGB1	1	1.447	2.313	1.099	1.214	1.314
20	junctional adhesion molecule 2	JAM2	1	0.758	0.588	0.779	0.222	0.604
21	junctional adhesion molecule 3	JAM3	1	0.493	0.581	2.121	0.599	1.040
22	lymphoid enhancer-binding factor 1	LEF1	1	0.841	0.847	0.878	0.883	1.048
23	mixed-lineage leukemia translocated to, 4	MLLT4	1	0.849	0.995	0.883	1.032	1.079
24	metadherin	MTDH	1	1.035	1.439	1.073	1.037	1.333
25	myosin, light chain 12 A, regulatory	MYL12A	1	2.014	2.305	2.106	0.595	0.331
26	myosin, light chain 12B, regulatory	MYL12B	1	0.506	0.601	1.095	1.044	1.281
27	myosin, light chain 6B	MYL6B	1	1.106	1.161	1.052	2.316	2.409
28	myosin, light chain 9, regulatory	MYL9	1	0.764	0.489	0.797	0.890	0.747
29	NK2 homeobox 1	NKX2-1	1	1.561	0.471	0.417	0.239	0.421
30	occludin	OCLN	1	0.966	1.177	1.009	0.638	0.619
31	poliovirus receptor-related 1	PVRL1	1	0.535	0.585	1.155	1.224	1.195
32	snail homolog 1	SNAI1	1	0.999	0.816	1.356	1.472	1.277
33	Sp1 transcription factor	SP1	1	0.917	1.132	0.953	1.071	1.355
34	transcription factor 7	TCF7	1	0.543	0.622	1.125	0.642	0.899
35	transcription factor 7-like 1	TCF7L1	1	1.830	0.975	0.453	0.514	1.065
36	transcription factor 7-like 2	TCF7L2	1	1.009	1.145	1.101	1.190	1.370
37	tight junction protein 1 (zona occludens 1)	TJP1	1	1.008	1.252	1.027	1.139	1.253
38	tight junction protein 2 (zona occludens 2)	TJP2	1	0.877	1.030	0.916	1.037	0.868
39	tight junction protein 3 (zona occludens 3)	TJP3	1	2.026	1.332	1.091	1.686	1.294
40	WNK lysine deficient protein kinase 4	WNK4	1	1.036	1.271	1.068	0.967	1.309

N/A: no amplification was observed. T-84 cells were pretreated (5 h) with 5-ASA (5 mM) or AZTP (10 μM) followed by TNF-α (10 ng/ml) treatment overnight (18 h).

To further examine the localization of epithelial junctional proteins, immunofluorescence was performed on T-84 monolayer for representative proteins of cell-cell contacts: E-cadherin (AJs), occludin (TJs) and desmoglein-2 (desomosomes). Untreated T-84 monolayer exhibited intact tight junctions. TNF-α treatment, however, resulted in internalization of all junctional proteins; with disruption of TJs, AJs and desmosomes (Fig. 1D). Pretreatment with 5-ASA and AZTP was protective and maintained membranous localization of proteins. We also examined TNF-α-induced NFκB activation and effect of these compounds on the nuclear translocation of p65 (RelA). TNF-α-induced increased nuclear p65 was mitigated by both 5-ASA and AZTP pretreatment (Supplementary Fig. 1E).

Intestinal alkaline phosphatase (ALPI) is expressed throughout gastrointestinal tract in apical brush border and plays an important role in reducing inflammation and intestinal permeability. Moreover, its levels are reduced in IBD^30^. Therefore, we examined the expression of by qRT-PCR as a marker of cellular differentiation which improves barrier function (Fig. 1E). TNF-α treatment reduced ALPI, whereas only 5-ASA induced its expression in the presence of TNF-α. Altogether, these data indicate that 5-ASA as well as AZTP facilitate membranous restoration of junctional proteins and suppress the effect of TNF-α to maintain epithelial integrity. Moreover, 5-ASA promotes cellular differentiation under proinflammatory conditions.

### Effect of 5-ASA and AZTP on epithelial barrier restoration in intestinal organoids

**Figure 2:**
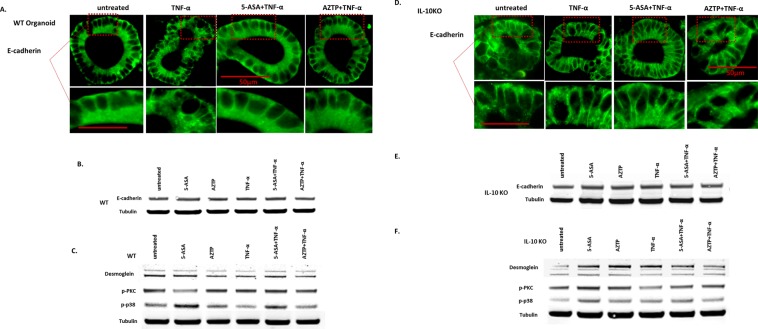
Molecular effects of 5-ASA and AZTP on epithelial barrier in intestinal organoids. E-cadherin expression in organoids pretreated with 5-ASA or AZTP in the presence or absence of TNF-α (10 ng/ml). Small intestinal organoids isolated from WT (**A**) or IL-10 KO (**D**) mice were pretreated with 5-ASA; 5 mM or AZTP 10 μM (5 h) followed by TNF-α treatment (10 ng/ml, overnight; 16 h). E-cadherin was visualized using Alexa Fluor 488 secondary antibody as described in methods. Image magnification 400x. Scale 50 μm. Western blot analysis of organoids from WT (**B**,**C**) and IL-10KO mice (**E**,**F**). α-tubulin was used as loading control. Images were acquired on Odyssey imaging system (LI-COR). Additional information on Western blots is available in the supplementary data. Organoids from multiple wells were pooled for the analysis.

Changes in E-cadherin recycling alter the integrity of AJs; and membranous E-cadherin is critical in the formation of AJs. We utilized tissue sections from wild type (WT) mice and IL-10 deficient mice (IL-10KO) with and without acute DSS colitis to examine E-cadherin expression. Non-inflamed crypts from WT mice exhibited membranous E-cadherin. Interleukin 10 knock out (IL-10KO) mice, which spontaneously develop enterocolitis, showed crypt elongation and aberrant expression of E-cadherin (Supplementary Fig. 2). Upon induction of colitis via DSS, the membranous expression of E-cadherin was disorganized, with cytoplasmic accumulation that was markedly increased in IL10KO mice. This conforms to previously reported loss of epithelial barrier in IL-10KO mice, and in intestinal inflammation^31^. To further assess the effect and mechanism of 5-ASA and AZTP on epithelial barrier, the *ex-vivo* intestinal organoid model was employed. IL-10KO organoids could represent a model of impaired barrier function, however, *ex vivo* effects of proinflammatory cytokines on IEC from these mice has not been examined before. Both WT and IL-10KO organoids were treated with TNF-α, in the presence or absence of 5-ASA and AZTP. TNF-α treatment resulted in internalization and abnormal distribution of E-cadherin in WT organoids, mimicking the acute DSS colitis model (Fig. 2A). Both 5-ASA and AZTP treatment restored membranous expression of E-cadherin. Untreated IL-10 KO organoids exhibited aberrant E-cadherin expression (disrupted membranous expression, diffused cytoplasmic expression), as was observed in the mouse tissue, and was not altered further upon TNF-α treatment (Fig. 2D). Although, AZTP treatment improved E-cadherin distribution, the effect was more pronounced upon 5-ASA treatment. Western blot analysis showed no change in total E-cadherin (Fig. 2B,E), supporting the immunofluorescence data that these drugs primarily affect re-distribution of proteins onto the surface. Desmoglein-2 expression was reduced by TNF-α in WT organoids, which was recovered by 5-ASA but not AZTP. However, in IL-10KO organoids, desmoglein-2 expression was increased in all treatment groups (Fig. 2C,F). The increased expression of desmoglein-2 was associated with activation of p38MAPK pathway, as previously reported^32^. This effect was more prominent upon 5-ASA treatment. Among other pathways implicated in the regulation of epithelial barrier, PKC activation was not altered by TNF-α in WT organoids, however, its levels were reduced in IL-10KO (Fig. 2C,F). Both 5-ASA and AZTP induced its expression in IL-10KO mice.

### Effect of 5-ASA and AZTP on wound healing

**Figure 3:**
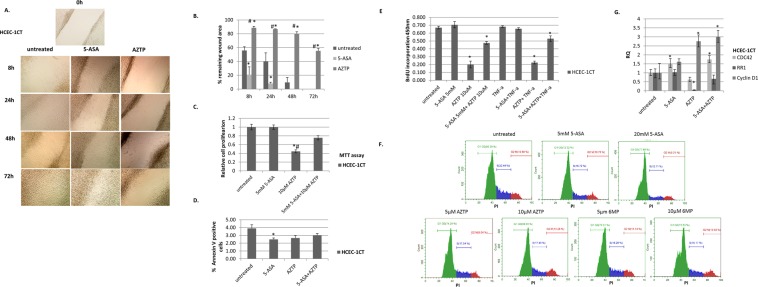
5-ASA promotes wound healing. (**A**,**B**) *In vitro* scratch assay on HCEC-1CT monolayer. Closure of scratch (wound healing) was observed under inverted microscope and pictures were taken at different time points indicated. Image magnification 100x. Pictures are representative of three independent experiments. Quantification was performed on two images. ANOVA with Tukey’s multiple comparisons test was performed (**C**) MTT based cell proliferation assay (48 h) in the presence or absence of 5-ASA (5 mM), AZTP (10 μM) or in combination was performed (n = 4) and repeated three times. (**D**) Measurement of apoptosis in HCEC-1CT by Annexin V staining after 24 h treatment with the compounds. Assay was performed in biological triplicates and repeated twice. p value (≤0.05), * con vs treatment; # 5-ASA vs AZTP. Kruskal-Wallis Test with Dunnet post hoc analysis was performed (**E**) BrdU incorporation in HCEC-1CT in the presence or absence of TNF-α (10 ng/ml) and 5-ASA (5 mM) or AZTP (10 μM). p value (≤0.05), * con vs treatment. ANOVA with Dunnett’s multiple comparisons test was performed (**F**) Dose dependent response of 5-ASA and AZTP (24 h) on cell cycle measured by flow cytometry using PI staining. (**G**) qRT-PCR analysis of genes for cell migration (CDC42) and proliferation (RR1, cyclin D1). p value (≤0.05), * con vs treatment. Dunnett’s multiple comparisons test was performed.

Healing of epithelial lesions, such as mucosal ulcers, is vital for disease remission in IBD. To elucidate the mechanism of 5-ASA and AZTP on wound healing, an *in vitro* scratch assay was performed on normal diploid HCEC-1CT monolayer. Untreated and 5-ASA treated wound began to heal (filling of gaps) at an early time point with complete gap filling within 72 h. This is indicative of normal cell proliferation, migration, and spreading (Fig. 3A,B). In AZTP treated monolayers, gap filling was delayed and sluggish. Upon injury, cells migration to the eroded area is facilitated by activation of cellular processes promoting cell proliferation, survival, migration, differentiation and polarization. A significant growth inhibitory effect was observed upon AZTP treatment, unlike 5-ASA. A combination of 5-ASA and AZTP was also used to test if 5-ASA could counteract the inhibitory effect of AZTP. 5-ASA did not alter cell proliferation but counteracted inhibitory effects of AZTP (Fig. 3C). 6MP exhibited similar effects like AZTP (Supplementary Fig. 3A,B). Effect of 5-ASA or AZTP on apoptosis in HCEC-1CT was further analyzed by Annexin V staining (Fig. 3D). Small fraction (~4%) of untreated HCEC-1CT was apoptotic. However, neither treatment increased apoptosis, and would rather reduce the %apoptotic cells. This indicates that both 5-ASA and AZTP promote cell survival in normal colon epithelial cells. To assess the effect of TNF, cell proliferation was measured by BrdU incorporation (Fig. 3E). As was observed in MTT assay of cell viability (Fig. 3C), AZTP inhibited BrdU incorporation indicating inhibition of cell proliferation. TNF treatment did not interfere with cell proliferation in HCEC-1CT. 5-ASA, nonetheless, partially counteracted the inhibitory effect of AZTP (Fig. 3C,E).

Next, the effect of these compounds on cell cycle distribution was analyzed upon treatment of HCEC-1CT with different doses of 5-ASA and AZTP and 6MP (Fig. 3F, Supplementary Fig. 3C). HCEC-1CT accumulated in G1 phase with 5-ASA treatment whereas both AZTP and 6MP showed reduced S phase in a dose dependent manner. This data corroborated with BrdU incorporation (Fig. 3E). To further investigate the growth inhibitory effect of AZTP, ribonucleoside-diphosphate reductase (RR1; an enzyme, crucial for deoxyribonucleotide production for DNA synthesis in S phase of dividing cells) mRNA expression was examined by qRT-PCR (Fig. 3G). A marked reduction in RR1 was observed upon AZTP treatment and thereby explained inhibition of cell proliferation. Combination of AZTP with 5-ASA counteracted the inhibitory effect of AZTP, supporting cell proliferation data. Cyclin D1 (CCND1) which is required for G1 to S transition, was increased upon AZTP (Fig. 3G). A reduction in cyclin D1 is required for DNA synthesis in S phase^33^. Since AZTP stabilizes cyclin D1 and inhibits RR1 expression in HCEC-1CT, this could impede cell growth. Interestingly, 5-ASA in combination with AZTP did not reduce cyclin D1 expression. Rho-GTPase CDC42 mRNA expression was also analyzed for its role in cell migration. A strong inhibition of CDC42 was observed upon AZTP treatment. Overall, these observations suggest growth inhibition of normal colon epithelial cells by AZTP, which is partially counteracted by 5-ASA. Inefficiency of wound healing by AZTP compared to 5-ASA could be attributed to its anti-proliferative and anti-migratory effects through inhibition of RR1 and CDC42 as well as delay in cell cycle progression.

### Effect of 5-ASA and AZTP on intracellular ROS production and senescence

**Figure 4:**
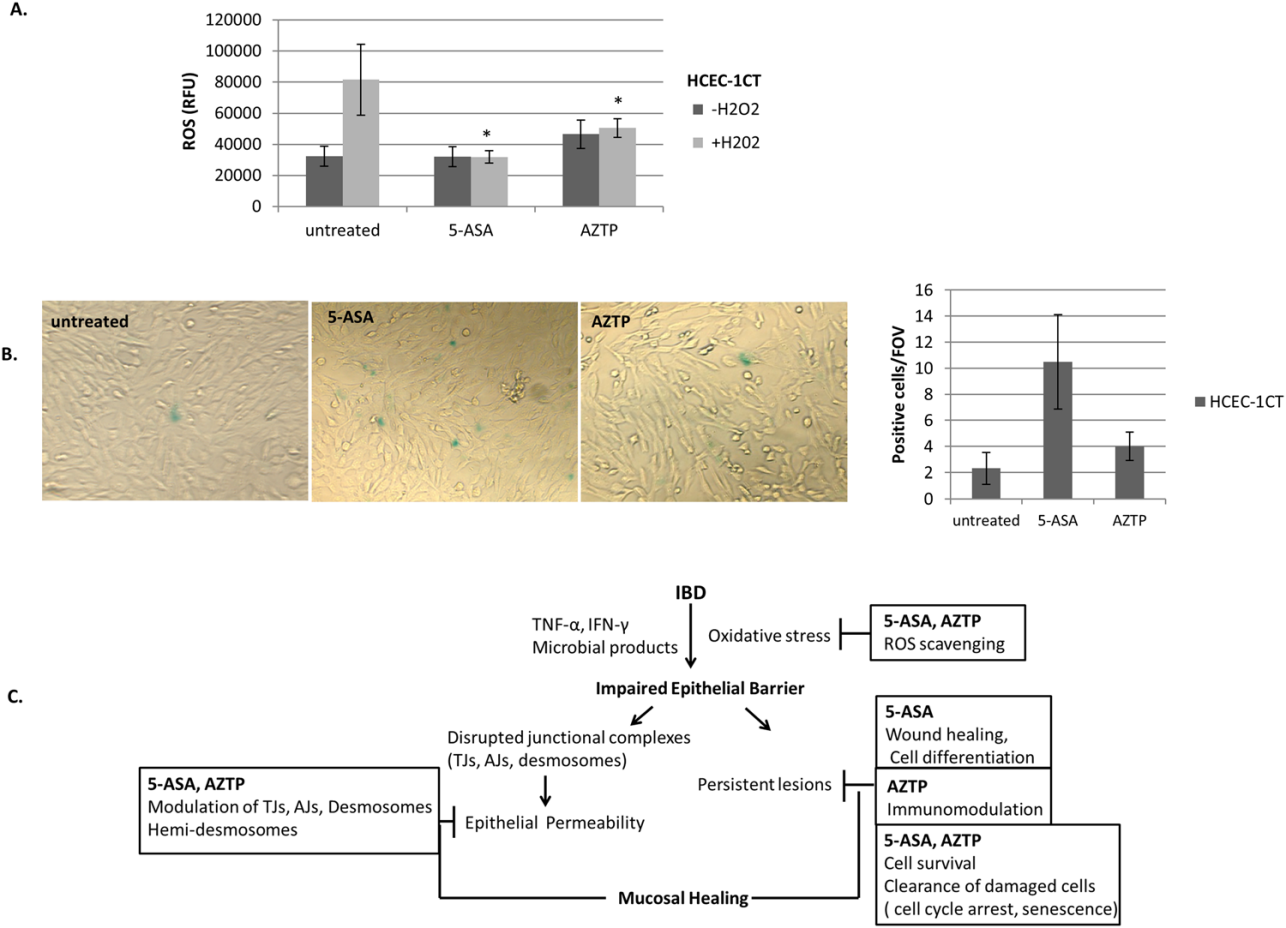
Cytoprotective effects of 5-ASA and AZTP (**A**) DCFDA based fluorescence assay for the measurement of intracellular ROS upon treatment with 5-ASA (5 mM) or AZTP(10 μM) in the presence or absence of H_2_O_2_ (100 μM, 1 h). n = 3; p value (≤0.05), *vs H_2_O_2_. Kruskal-Wallis Test with Dunnet post hoc analysis was performed. (**B**) Senescence associated β-galactosidase activity in HCEC-1CT. Positive (blue) cells were counted under inverted microscope (100x). Positive cells counted per field of view (FOV). 5 FOV were counted per sample. Experiment was performed in biological duplicates. (**C**) Model depicting molecular effects of 5-ASA (mesalamine) and AZTP (azathioprine) in mucosal healing.

In IBD, excessive oxidative stress induces mucosal injury and impairs epithelial barrier function and restitution. The reactive oxygen species (ROS) scavenging effect of 5-ASA is well known, however the effect of either of these compounds on normal colon epithelial cells is not reported. Here, intracellular ROS production was measured in HCEC-1CT treated with 5-ASA or AZTP in the presence and absence of oxidative stress (H_2_O_2_). A slight increase in intracellular ROS was observed in AZTP-treated cells, however both 5-ASA and AZTP exhibited ROS scavenging effects upon treatment with H_2_O_2_ (Fig. 4A). Cellular senescence, an intrinsic mechanism to counteract expansion of damaged cells^34^, was also examined for these compounds. Non-replicating, metabolically active cells stain positive (blue color) for senescence-associated β-galactosidase activity. Compared to untreated cells, 5-ASA and AZTP treatment showed a trend for an increase in the number of senescent cells in HCEC-1CT (Fig. 4B).Overall, these observations provide insight on physiological effects of 5-ASA and AZTP on colonic epithelium which might affect cellular response under stress (Fig. 4C).

## Discussion

In IBD, mucosal healing is a key parameter to assess the efficacy of treatment, and a predictive factor for sustained clinical remission. Therapeutics in IBD affects structural epithelial integrity, contributing to mucosal healing, as indicated by endoscopic clinical remission. A better understanding of drug mechanisms may provide insight on important pathologic pathways in IBD. Here we examined the molecular mechanisms of 5-ASA and AZTP using normal diploid colon epithelial cells and intestinal organoids, unlike previous studies primarily utilizing colorectal cancer cells. This study demonstrates that both 5-ASA and AZTP decrease epithelial paracellular permeability through rearrangement of junctional proteins such as occludin, E-cadherin and desmoglein-2 (Fig. 1D). Without inducing cell apoptosis, the compounds restore epithelial barrier in normal epithelial cells through induction of pro-survival pathways, senescence, and modulation of cell cycle progression. Unlike 5-ASA, AZTP exhibited inhibitory effect on cell proliferation, and was not effective in wound healing (Fig. 3). This response of AZTP was attributed to inhibition of Rho-GTPases such as CDC42, affecting cell migration and re-epithelization; as well as inhibition of DNA synthesis in S phase, due to suppression of the enzyme RR1, and stabilization of cyclin D1. The data also demonstrates that 5-ASA and AZTP, when used in combination, do not show additive effect. It was observed that 5-ASA counteracted AZTP activity, such as inhibitory effects on cell proliferation (Fig. 3C,E). Loss of membranous expression of E-cadherin observed during intestinal inflammation was also reflected in organoids upon TNF-α treatment; and this was recovered by both 5-ASA and AZTP. Our data substantiated the role of p38MAPK pathway in the maintenance of epithelial barrier. This pathway was downregulated by TNF-α in IL-10KO and WT organoids, which was prevented by 5-ASA. Altogether, these data demonstrate shared as well as differential pathways of 5-ASA and AZTP that contribute to the maintenance of epithelial barrier function.

Apical junctions comprising of TJs and AJs are disrupted upon loss or internalization of complex forming proteins such as E-cadherin (AJs) or occludin (TJs)^35^. Recruitment of TJ proteins to the apical lateral membrane is the key event in the closure of the paracellular space. However, TJ assembly is largely dependent on AJ formation^36^. It has been previously shown that 5-ASA activity restores membranous localization of E-cadherin and β-catenin^20^ and reduced barrier permeability by IFN-γ^37^. Thiopurines, however, are mostly studied as immunomodulators, and knowledge about their molecular effects on epithelial cells is lacking. Membranous restoration of AJ and TJ proteins by AZTP was not reported previously. Using PCR arrays (Fig. 1B,C), we identified novel targets of 5-ASA and AZTP that might contribute to epithelial barrier integrity. For example, phosphorylation of myosin light chain (MYL) is one of the key events for increasing TJ permeability^38^; and both 5-ASA and AZTP treatment reduced MYL12A expression. Of the caudal-related homeobox transcription factor family (CDX), CDX2 showed increased expression particularly upon 5-ASA. This gene is a critical regulator of intestinal homeostasis as well as mucosal healing, and is downregulated in UC^39^. Notably, claudin 11 (CLDN11) and JAM-2 and -3 expression were differentially regulated in TNF-α treated cells; however, their role in intestinal TJs remains elusive. Nevertheless, our data warrants validation of these targets at protein levels to elucidate further cellular mechanisms that remain largely unknown in intestinal epithelia.

Desmoglein-2 is the only isoform expressed by IEC forming desmosomes important in the formation of epithelial barrier. A reduced activity of p38MAPK and desmoglein deficiency was found to be associated with impairment of epithelial barrier and its reformation^32^. Our data in IL-10KO organoids corroborated this finding. Both desmoglein and phospho-p38MAPK expressions were reduced, indicating impaired barrier function in these mice^31^. A similar effect was observed upon TNF-α treatment of WT organoids, substantiating the role of p38MAPK in the regulation of epithelial junctions. 5-ASA was effective in activating p38MAPK, indicating its action through modulation of stress response pathways. AZTP, however, exhibited milder effects only in IL-10KO organoids.

Mucosal lesions in IBD elicit the wound healing response; a dynamic process of tissue remodeling that involves a fine balance of cell proliferation, migration, apoptosis, and differentiation. Our data on HCEC-1CT demonstrated that 5-ASA but not AZTP was efficient in re-epithelialization of the wound. This could be attributed to the inhibitory activity of AZTP on both growth and cell migration, and inhibition of Rho-GTPase CDC42 which coordinates cell proliferation, migration, polarity and differentiation^40^.

The effect of 5-ASA on S phase arrest has already been demonstrated in CRC cells^41^. In contrast, a dose dependent accumulation of cells in G1 was observed HCEC-1CT treated with 5-ASA. AZTP reduced transition of cells to S phase and G2/M; and this could be a consequence of cyclin D1 stabilization and reduction of RR1, which impede DNA synthesis. Variations in cyclin D1 levels are observed through the cell cycle and its suppression is requisite for DNA synthesis in S phase^33^. An induction of senescence was also observed upon both 5-ASA and AZTP treatments, which might contribute to cell survival and tissue repair. Cellular senescence, an state of metabolically active yet non-replicating cell, is implicated in wound healing and other aspects of tissue homeostasis^42^. Altogether, delayed cell cycle progression and cellular senescence could contribute to either repair or clearing of damaged cells, improving epithelial integrity. Oxidative stress is one critical factor in causing mucosal injury and thereby impairing epithelial barrier in chronic gut inflammation^9^. The ROS scavenging effect of these compounds indicate their cytoprotective role on normal intestinal epithelial cells under oxidative stress.

In the setting of chronic disease, long term efficacy of therapeutics is critical and warrants mechanistic insight to facilitate understanding of disease pathogenesis and to yield novel therapeutic approaches. Maintenance therapies could provide better insight about underlying mechanisms of mucosal healing and both 5-ASA and AZTP are effective as maintenance therapy in UC. The rationale of immunosuppressive drugs in the treatment of IBD was based on the observations of immune based mechanisms. However, this study suggests direct effects of AZTP on IEC which play a pivotal role in the maintenance of the intestinal structure and function. The mechanistic findings (Fig. 4C**)** of this study underscore both benefits and limitations of the currently used drugs and identify potential mechanisms and targets of functional epithelial barrier.

## Materials and Methods

### Cell culture and Reagents

T-84 cells (from ATCC) were cultured in Dulbecco’s Modified Eagle Medium-F12 (Gibco, ThermoFisher Scientific), which was supplemented with 10% fetal bovine serum (Biochrom, Berlin, Germany) and penicillin-streptomycin solution (Gibco, ThermoFisher Scientific). The cell line is derived from human colonic carcinoma and is a widely used model system to study paracellular permeability. Upon confluence, these cells differentiate spontaneously to form polarized monolayers with well-formed tight junctions^43,44^. Primary human colon epithelial cells HCEC-1CT cells (obtained from Jerry W. Shay and Andres I. Roig, University of Texas, Dallas), were cultured as previously described^45^. These cells are immortalized colonic epithelial cells derived from human colon biopsies. The cells express stem cell markers and differentiate *in vitro*. Cells were cultured in Primaria flasks (Becton Dickinson, Germany) at 37 °C, 5%CO_2_ and 100% humidity. Proinflammatory mediators used were human recombinant IFN-γ (100U/ml), LPS (10 ng/ml; eBioscience, Austria) and TNF-α (10 ng/ml; Miltenyi Biotec, Germany).

### Intestinal organoids

Small intestinal and large bowel organoids (LBO) were isolated from C57BL/6J wild type (WT) and IL-10−/− (IL-10 KO) mice and maintained as previously described by Sato *et al*.^46^. Organoids were cultured in 24-well plates in 50 µl Matrigel (BD Bioscience, Franklin Lakes, NJ) droplets and 500 µl organoid culture medium consisting of murine recombinant noggin (Peprotech), epidermal growth factor (Invitrogen) and R-spondin 1 (R&D Systems) or 50% conditioned Wnt, R-spondin1, noggin-medium (LBOs). Organoids were passaged (1:3) every 3–5 days.

### 5-ASA and AZTP

Mesalamine (>99.9% pure; a generous gift from Shire Inc., Eysins, Switzerland) was dissolved in the culture medium at 5 mM final concentration for most experiments (unless indicated; pH adjusted to 7.2 with NaOH) as described earlier^29^. This concentration of 5-ASA is a physiologically relevant dose (in lower range) used in IBD therapy^47^. Azathioprine was purchased from Sigma and used at 10 μM final concentration (unless otherwise indicated). This concentration of AZTP has been previously shown to result in intracellular 6-TG levels that are comparable to those reported in leukocytes of patients with Crohn’s disease receiving long-term thiopurine treatment^15,48,49^. The drug pretreatment was at least 5 h (before TNF-α treatment).

### Paracellular Permeability

T-84 cells (1 × 10^5^) were plated in duplicates on 24-well polystyrene Transwells (0.4-μm pore size; Costar, Corning, NY, USA). Cells were treated when the trans-epithelial electrical resistance (TEER), (measure of epithelial barrier integrity), was over 1,000 ohmscm^2^ as measured with an epithelial voltohmmeter (World Precision Instruments, Sarasota, FL, USA). Pretreatment of 5-ASA (5 mM) or AZTP (10 μM) was apical. Cytokines were added to either apical (TNF, LPS) or basolateral side (IFN-γ) of transwells. Paracellular permeability was determined (24 h following treatments) by apical to basolateral flux of 10-kDa fluorescein isothiocyanate- labeled dextran (FITC-dextran, Sigma). From the basolateral side, 200 μL sample was transferred to a black 96-well plate (Costar, Corning, NY, USA) and the fluorescence was measured using a Chameleon Counter (HVD Life Sciences) at 485 nm/535 nm.

### Immunofluorescence analysis

*T-84 monolayer*: Cells were fixed in methanol and immunostaining was performed using antibodies against occludin (Sigma), E-cadherin (Clone 36; BD Bioscience), desmoglein-2 (Abcam), hemi-desmosome subunit ITGA6 (CD49f, GoH3 monoclonal: BD Bioscience) and p65/RelA (Santa Cruz Biotechnology). For protein visualization, AlexaFluor 488 and 568 antibodies (Invitrogen, Thermo Fisher Scientific) were used. Nuclear staining was performed using DAPI with Vectashield (Vector laboratories) for mounting. *Intestinal Organoids*: Small intestinal organoids were fixed in 4.5% formaldehyde solution overnight at 37 °C and embedded in Tissue-Tek optimum cutting temperature (O.C.T.) Medium (Sakura Finetek, The Netherlands) and kept at −80 °C. Cryosections were treated with blocking solution followed by primary antibody (E-cadherin; BD Bioscience) incubation (overnight 4 °C) and proteins were visualized using secondary antibody (anti- mouse Alexa Fluor 488; Invitrogen ThermoFisher Scientific). Slides were mounted with Mowiol (Calbiochem). Images were recorded at 400x magnification on an Olympus BX51 microscope, using Cellsens dimension life science imaging software (Olympus; Hamburg, Germany) and processed with Adobe Photoshop (Adobe, Mountain View, CA).

### Quantitative real-time PCR

RNA was isolated using TRIzol reagent (Life Technologies). cDNA synthesis was carried out using Thermoscript RT-PCR System (Invitrogen) according to manufacturer’s protocol. Quantitative RT-PCR was carried out in triplicates using Fast SYBR Green Master Mix with human primers (QuntiTect Primer assay, Qiagen: RRM2B (QT00074781); CDC42 (QT01674442); CCND1(QT00495285), API (QT00201957), GAPDH (QT01192646) and 36B4 (forward 5′ GCT TCA TTG TGG GAG CAG ACA 3′, reverse 5′ CAT GGT GTT CTT GCC CAT CAG 3′). All data were normalized to the endogenous control 36B4. Relative quantification of transcripts was calculated using the comparative Ct method. *Real-Time PCR array***:** Pathway Predesigned PCR array (Bio-Rad, Austria) was used for TJ (Cat#10025728) gene analysis of T-84 cells treated with TNF-α in the presence or absence of 5-ASA and AZTP. Data was analyzed with PrimePCR™ Analysis Software (Bio-Rad). The array contained 3 housekeeping genes (TBP, GAPDH and HPRT1) that were used for normalization of the data. Any Ct value >35 was considered to be negative. If the Ct value of the genomic DNA control was ≥30, then no genomic DNA was detectable. The software calculates the fold change based on ∆∆Ct method.

### Western Blotting

Large bowel organoids were pretreated with 5-ASA or AZTP followed by TNF-α treatment (overnight). Organoids were washed in PBS and resuspended in cell recovery solution (Corning, #354253) and incubated on ice (30–60 min). Organoid pellet was collected after depolymerization of Matrigel (300 × g, 5 min, 4 °C). Pellet was washed in PBS and lysed in RIPA buffer (30 min on ice) with intermittent vortexing. Supernatant was collected after centrifugation (13000 rpm, 10 min at 4 °C) and protein concentration was determined by Bradford method (Bio-Rad). 20 µg of protein samples in Laemmli sample buffer were separated by SDS–PAGE and immune-blotted onto a PVDF membrane. The protein bands were visualized with IRDye coupled anti-rabbit or anti-mouse antibodies (either or both mouse/rabbit; LI-COR) and scanned on Odyssey imager (LI-COR Biotechnology). Antibodies used were: ZO-1 (Invitrogen, Thermo Fisher Scientific), occludin, cleaved caspse3, p-p38MAPK, p-PKC (cell signaling); E-cadherin, integrin-α6 (BD Bioscience); PCNA, desmoglein-2 and α-tubulin (Abcam).

### Cell proliferation assay

Cell proliferation was evaluated after 48 h with a standard MTT assay. 10000 cells/well were seeded into 96-well plates. Cell proliferation was evaluated after 48 h with a standard MTT assay. Briefly, MTT (Thiazoyl Blue Tetrazolium Bromide; Sigma, M5655) reagent was freshly dissolved. 20 µl of the 5 mg/ml reagent was added to each well and incubated for 3 h at 37 °C in the dark. The media was removed and 150 µl of DMSO/ethanol solvent (1:1) was added per well. The plate was covered in aluminium foil and placed on a shaker for 15 min at 25 °C. The absorbance was measured on a microplate reader (Anthos 2010) at 570 nm with a reference filter set at 620 nm. Each measurement was performed in biological triplicates.

### Wound healing assay

An *in vitro* scratch assay was performed to measure the effect of 5-ASA and AZTP on wound healing. The HCEC-1CT monolayer (6-well plate) was scraped in a straight line to make a “scratch” with a p200 pipet tip. The debris was removed by washing the cells with 1 ml of the growth medium. Fresh medium with compounds (5 mM 5-ASA; 10 μM AZTP) was added and the plates were incubated at 37 °C in CO_2_ incubator. Pictures were taken periodically (0 h–72 h) and wound area was quantified using ImageJ software (National Institutes of Health, Bethesda, USA). The experiment was performed three times.

### Annexin V Staining and Cell cycle analysis

The apoptosis assay was performed using the Annexin V detection kit (eBioscience, Austria) in accordance with the manufacturer’s instructions. Cell cycle distribution was assessed through flow cytometric measurement upon propidium iodide staining (Sigma). Flow cytometry was performed on a Cell Lab Quanta SC flow cytometer (Beckman Coulter, Vienna, Austria) or a Cytoflex (Beckman Coulter, Vienna, Austria) and analyzed with respective machine software.

### BrdU Incorporation Assay

Quantification of newly synthesized DNA of actively proliferating cells was determined using BrdU Cell Proliferation Assay Kit (Millipore©). After addition of goat anti-mouse IgG-peroxidase conjugated secondary antibody, BrdU incorporation was determined using microplate reader set at dual wavelength of 450/550 nm (Anthos 2010).

### Intracellular ROS measurement

Intracellular reactive oxygen species (ROS) was measured by DCFDA (2′,7′–dichlorofluorescein diacetate) assay. 6000 cells/well were seeded in quadruplicate (96 well plate). 100 μM hydrogen peroxide (H_2_O_2_) was added in the wells (1 h) with and without pretreatment of the compounds: 5-ASA (5 mM), AZTP (10 μM) for 5 h. Subsequently, cells were washed twice with PBS and 100 µl of 10 µM DCFDA (in HBSS) was added and incubated for 30 min (37 °C in CO_2_ incubator). After washing the cells with PBS, 100 µl HBSS was added and cells were again incubated for 10 min. Fluorescence was measured using a Chameleon Counter (HVD Life Sciences); excitation 485 nm; emission 535 nm.

### Senescence-associated β-Galactosidase Cell Staining

β-galactosidase staining was performed as per manufacture’s protocol (Cell signaling Technology). The kit detects only β-galactosidase activity at pH 6 which is present only in senescent cells and is not found in pre-senescent, quiescent or immortal cells. Briefly, cell monolayer was washed with PBS after the treatment and fixed for 15 min. Staining solution was added and plate was incubated at 37 °C overnight. The blue cells were scored under the microscope at 100x magnification. At least five fields of view were scored.

### Statistics

GraphPad Prism 6.01 software (GraphPadSoftware) was used to analyze all data. Normally distributed data was analyzed using one-way ANOVA. Multiple comparisons were done using Bonferroni’s post hoc analysis. If data was not normally distributed, samples were compared using Kruskal-Wallis Test with Dunnet post hoc analysis. Two-way ANOVA with Tukey’s HSD-post hoc test was used for samples with two or more independent variables. P-values less than 0.05 were considered statistically significant.

### Ethical Considerations

Animal experiments were performed in accordance with the Austrian and European law, defined by the Good Scientific Practice guidelines of the Medical University of Vienna. The ethical approval was obtained for the experimental protocol (BMWF-66-009/0324-WF/V/3b/2016) from Bundesministerium für Wissenschaft, Forschung und Wirtschaft (BMWFW); Federal Ministry of Science, Research and Economy.

## Supplementary information

Supplementary Information
