# Supplementary material for: Mesalamine and azathioprine modulate junctional complexes and restore epithelial barrier function in intestinal inflammation
Source: Sci Rep. 2019 Feb 26;9:2842. doi: 10.1038/s41598-019-39401-0 (PMC6391397; doi:10.1038/s41598-019-39401-0)
Supplement: Supplementary file 1 — Supplementary Information [file 41598_2019_39401_MOESM1_ESM.pdf]

Supplementary Information

Mesalamine and azathioprine modulate junctional complexes and restore epithelial barrier function in intestinal inflammation

[1]Vineeta Khare, [1]Anita Krnjic, [1]Adrian Frick, [1]Christina Gmainer , [1]Mario Asboth, [1]Kristine Jimenez, [1]Michaela Lang, [1]Maximilian Baumgartner, [1]Rayko Evstatiev and [1*]Christoph Gasche

[1]Department of Internal Medicine III, Division of Gastroenterology and Hepatology, Medical University of Vienna, Vienna, Austria.

Supplementary Methods

Acute DSS-colitis

C57BL/6 mice (wild type; WT and IL-10 deficient; IL-KO) were induced with acute DSS colitis (1.7% DSS) for 5 days and euthanized (after 7 days). Intestines were prepared in Swiss-roll and paraffin embedded. Animal experiments were performed in accordance with the Austrian and European law, defined by the Good Scientific Practice guidelines of the Medical University Vienna.

Immunohistochemistry

Immunohistochemistry was performed on paraffin-embedded sections from mouse intestinal tissue. Sections were dried, dewaxed and rehydrated followed by blocking of endogenous peroxidase using 15% H_2O_2 in methanol for 10 min. Subsequent antigen retrieval was accomplished by boiling the sections in 10 mM citrate buffer (pH 6). After blocking, the primary antibody (E-cadherin, BD Bioscience) was added in for overnight at 4°C in a humidified chamber. The biotinylated secondary antibody was applied for 30 min at room temperature. The Avidin-Biotin-HRP complex (Vectastain) was added (30 min), and 3,3'-diaminobenzidine (DAB; Fluka) was utilized to visualize the staining. Nuclear counterstaining was achieved with hematoxylin. Images were recorded on an Olympus BX51 microscope.

Supplementary Figure 1

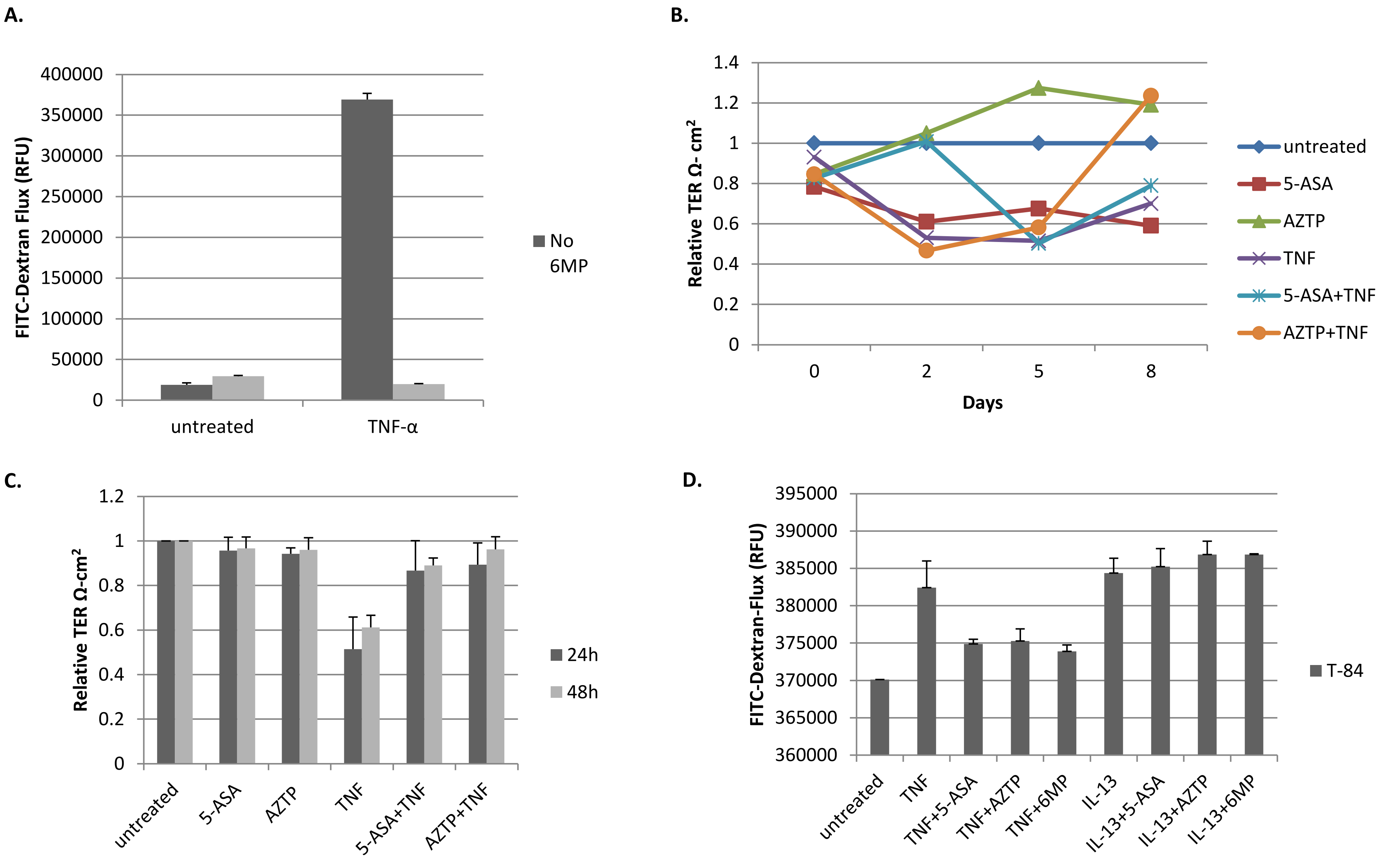

Effect of 5-ASA and Thiopurines on trans epithelial electrical resistance (TER) and paracellular permeability. A. FITC-dextran Flux was measured upon 6MP treatment (n=4) in the presence or absence of TNF-α. B. TER changes duringT-84 monolayer (n=2) formation was monitored in the presence of compounds and TNF-α. C.T-84 monolayer (n=2) was established and TER changes were measured upon different treatments. D. Effect of 5-ASA/ AZTP on paracellular permeability on a disrupted monolayer (n=2) upon TNF-α/ IL-13 treatment. 5-ASA (5mM); AZTP and 6MP (10µM); TNF-α (10ng/ml). Permeability assay was performed after 16-18 h post treatment. Error bars denote standard deviation.

E.

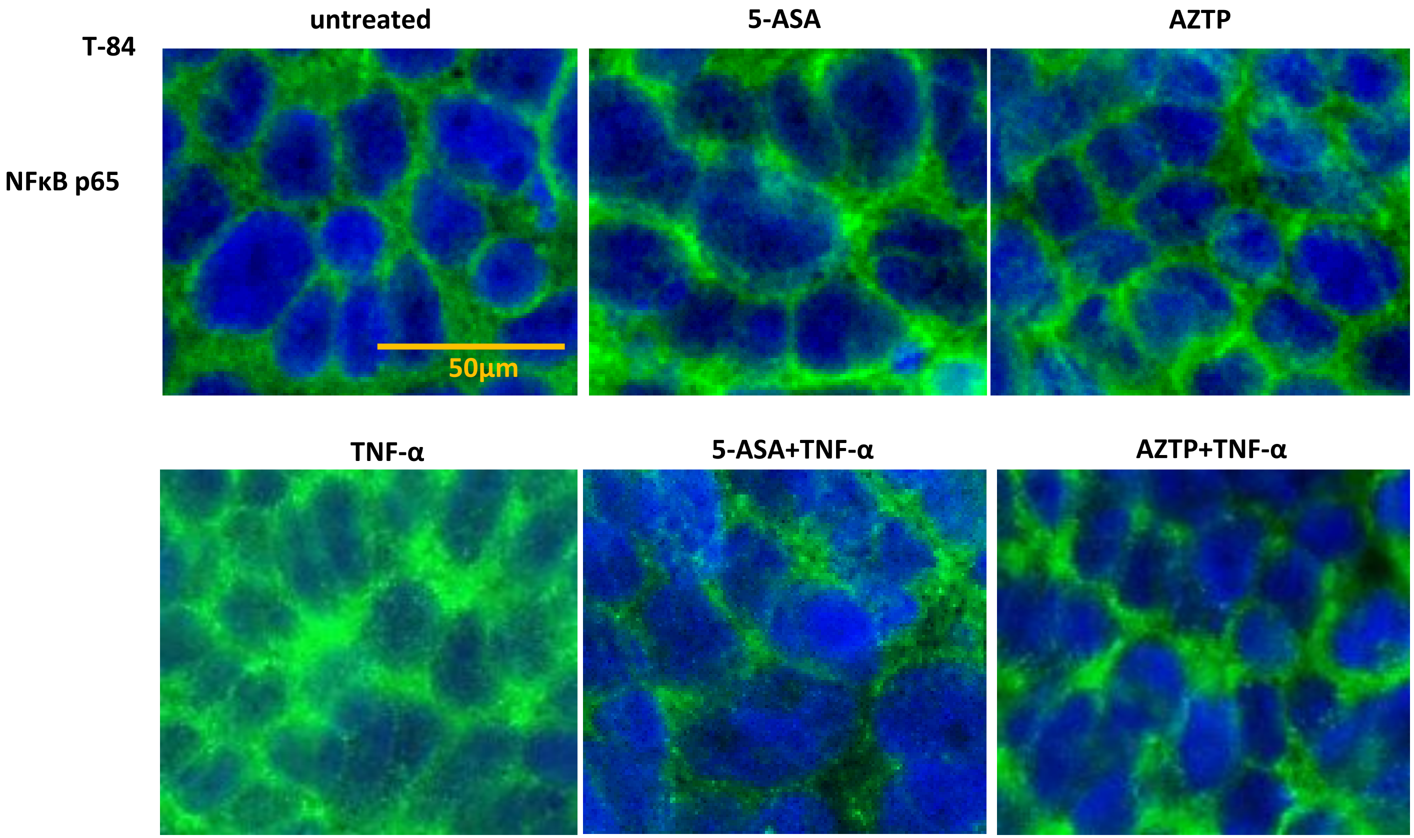

TNF-α induced NFκB signaling was examined by immunoflourescence using p65 (RelA) antibody. Nuclear translocation of p65 upon TNF-α was attenuated in 5-ASA or AZTP pretreated cells (n=2). Images were acquired at 400x magnification on an Olympus BX 51 microscope. Alexa Fluor 488 was used as a secondary antibody.

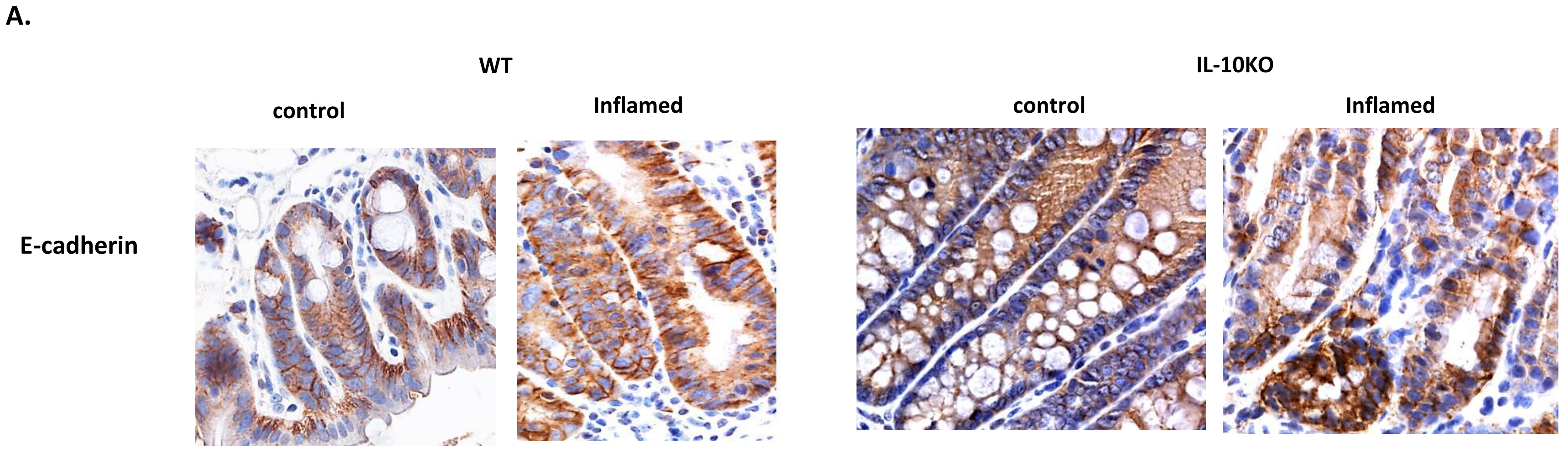

A. E-cadherin expression and localization using immunohistochemistry analysis on mouse tissue in wild type (WT) or IL-10 deficient (IL-10KO) mice. Mice were induced with acute DSS colitis as described in supplementary methods. Images are taken from colon sections representing normal (control) and Inflamed areas. Image magnification 200x.

A.

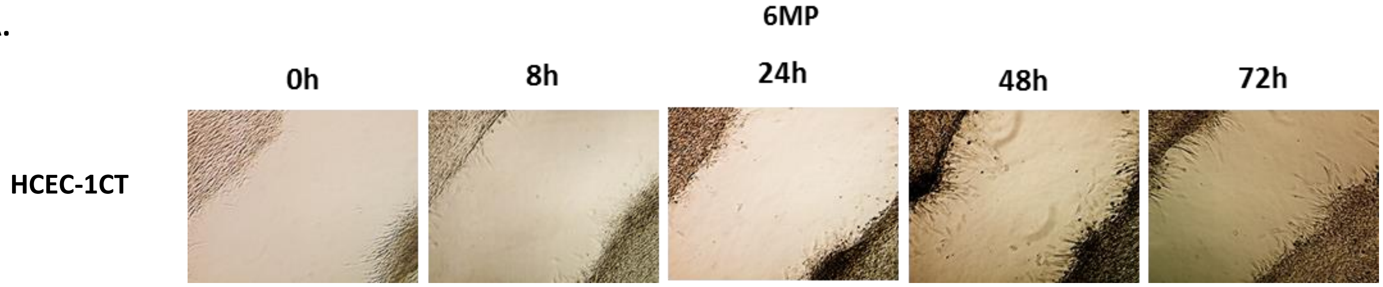

B.

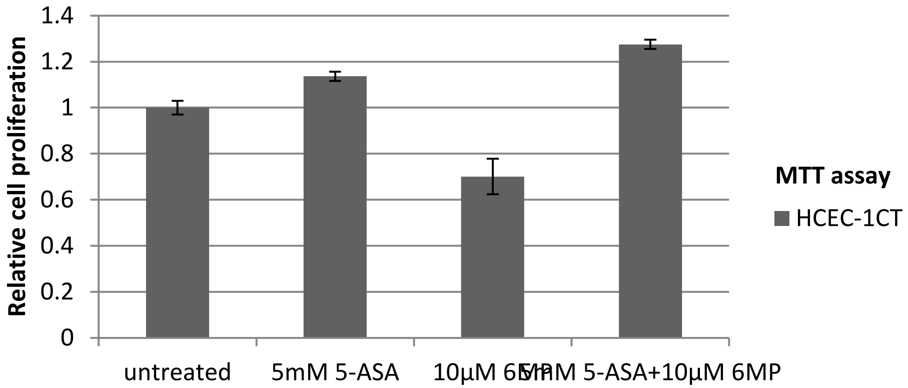

C.

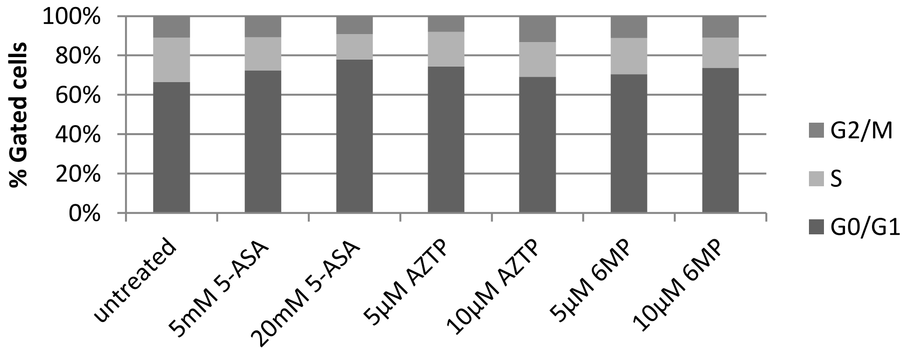

Effects of AZTP metabolite 6MP on HCEC-1CT. A. Wound healing assay scratch assay. 6MP(10µM) inhibited re-epithelization of the wound similar to AZTP. The assay was performed in biological duplicates. B. MTT assay for cell proliferation was performed after 48h of treatment (n=4). C. Graph showing % gated cells in FACS analysis using propidium iodide (PI) staining.

Supplementary Information

Western Blot Analysis of intestinal organoids isolated from WT and IL-10 KO mice

The protein bands were visualized with IRDye coupled anti-rabbit or anti-mouse antibodies (either or both mouse/rabbit; LI-COR) and scanned on Odyssey imager (LI-COR Biotechnology). Utilizing two different channels of detection (700nm and 800nm), multiple protein bands were visualized either together or in a sequential manner. The raw blots in B/W are provided. The images were processed in Odyssey software and cropped / enhanced using brightness and contrast/ rotation tools in Adobe Photoshop. Wherever quality was enhanced, it was applied to the entire image/protein band row. Only blots used in the final figures are shown. Some antibodies resulted in multiple bands. Bands representing expected size were used in the final figure. The size of the bands was determined using pre-stained protein marker ladder used while running the gels. The marker gives different patterns in the two color channels.

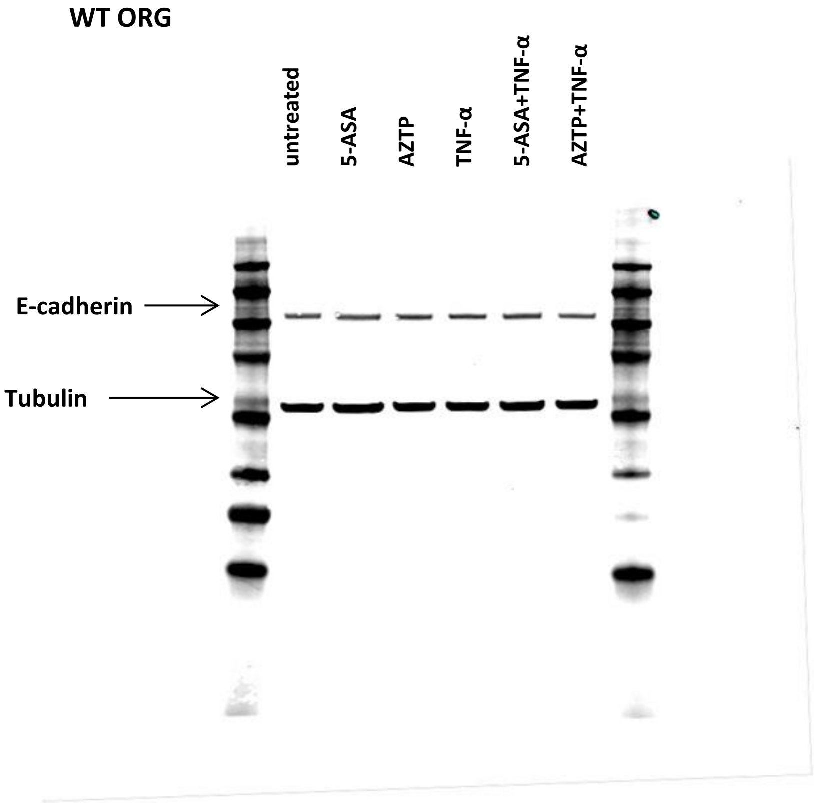

WT ORG
untreated
5-ASA
AZTP
TNF-α
5-ASA+TNF-α
AZTP+TNF-α
E-cadherin
Tubulin

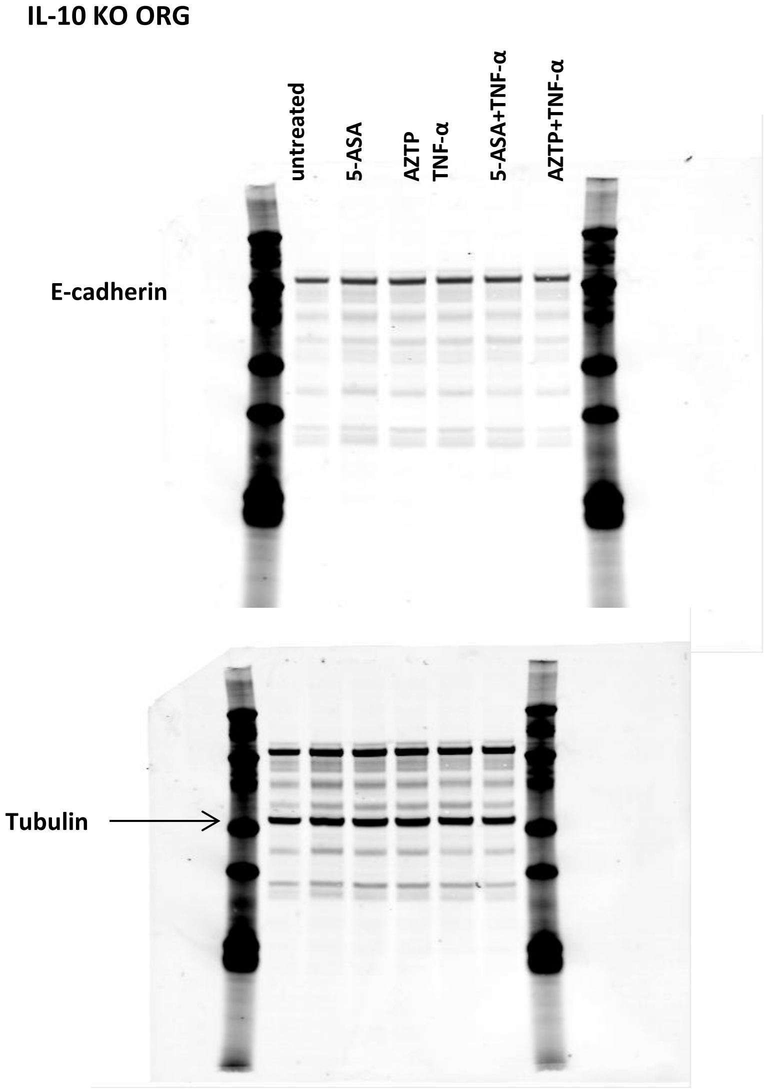

IL-10 KO ORG
untreated
5-ASA
AZTP
TNF-α
5-ASA+TNF-α
AZTP+TNF-α
E-cadherin
Tubulin

WT ORG

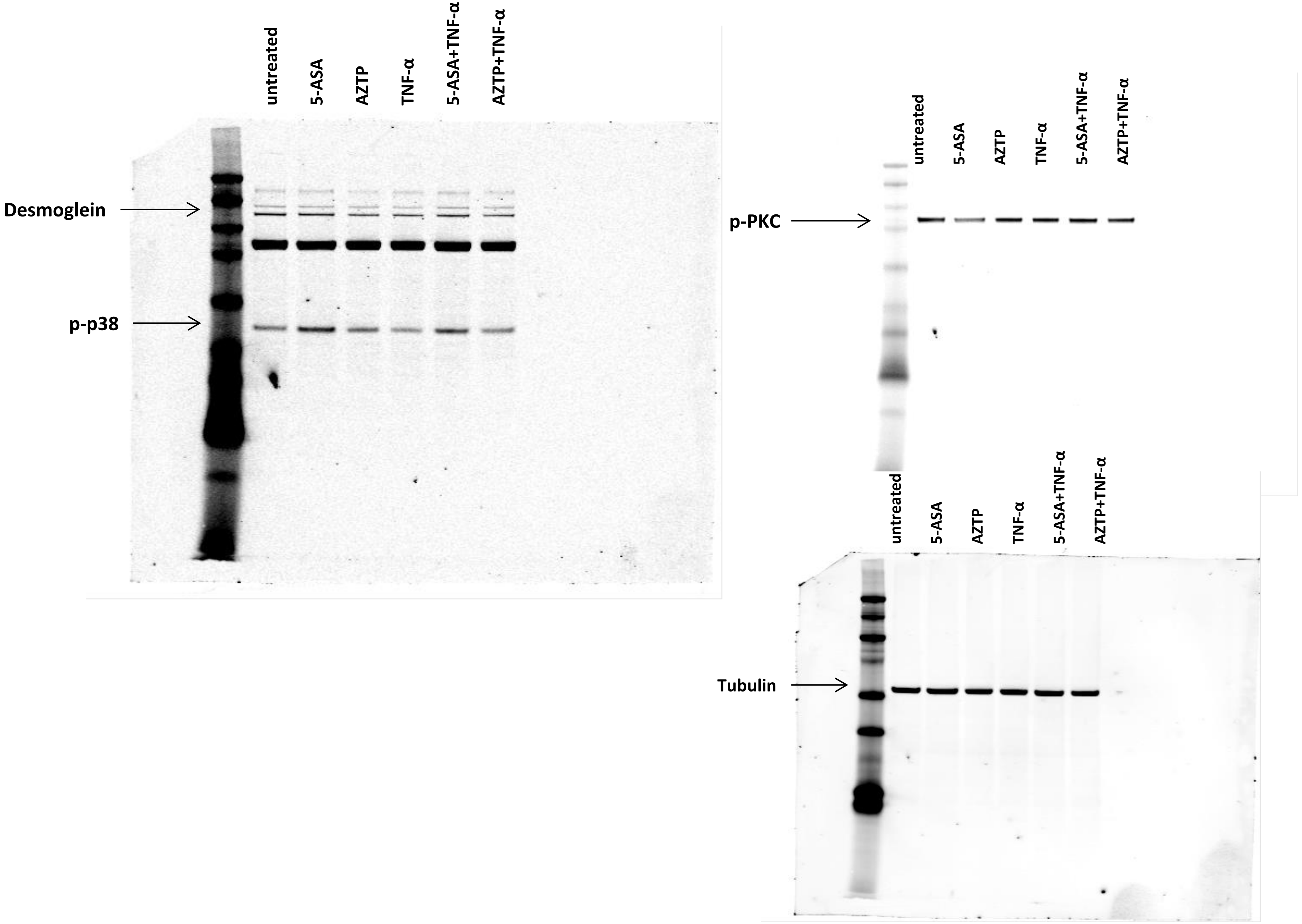
untreated
5-ASA
AZTP
TNF-α
5-ASA+TNF-α
AZTP+TNF-α
Desmoglein
p-p38
p-PKC
Tubulin

IL-10 KO ORG

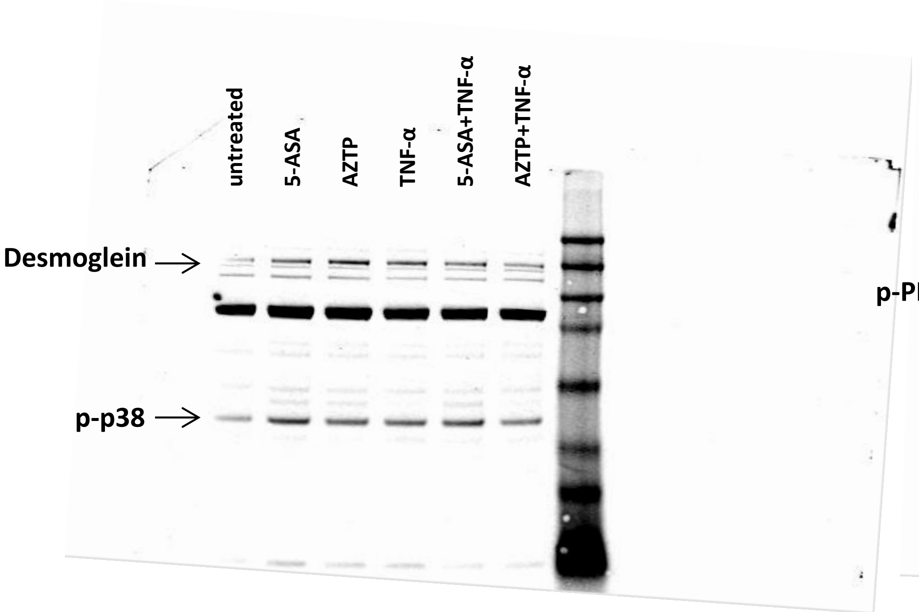
untreated
5-ASA
AZTP
TNF-α
5-ASA+TNF-α
AZTP+TNF-α
Desmoglein
p-p38

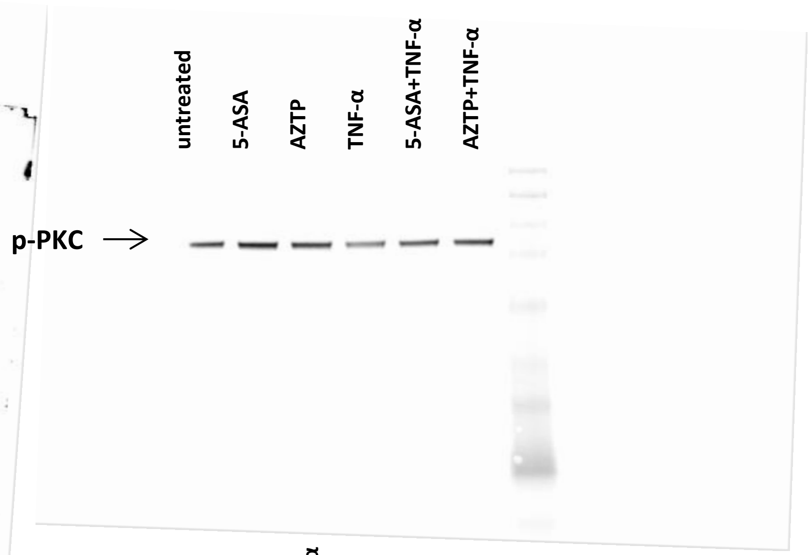
untreated
5-ASA
AZTP
TNF-α
5-ASA+TNF-α
AZTP+TNF-α
p-PKC

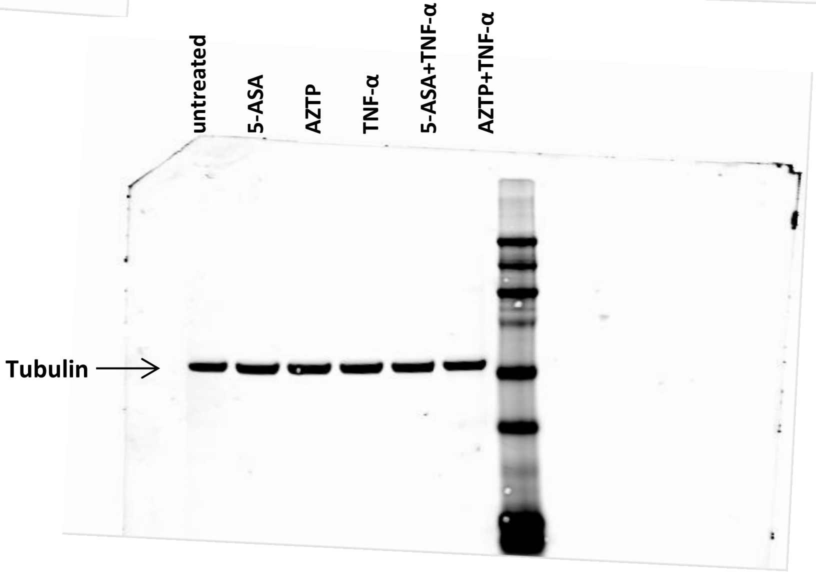
untreated
5-ASA
AZTP
TNF-α
5-ASA+TNF-α
AZTP+TNF-α
Tubulin

Secondary antibody (only) controls for the immunofluorescence

Organoid

T-84

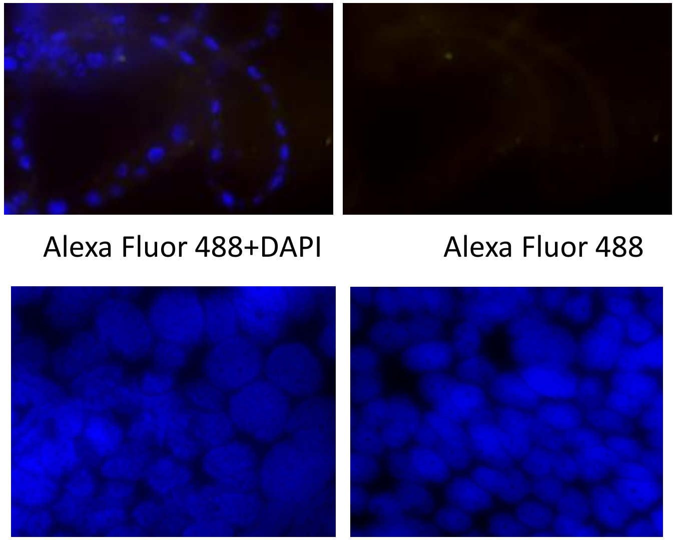

Alexa Fluor 488+DAPI

Alexa Fluor 488

Alexa Fluor 488 +DAPI

Alexa Fluor 568+DAPI
